# Adenosine A3 Receptor and Its Potential Role in Cancer Treatment: A Narrative Review

**DOI:** 10.7759/cureus.93950

**Published:** 2025-10-06

**Authors:** Joseph V Pergolizzi, Jo Ann K LeQuang, Mark H Coleman

**Affiliations:** 1 Pain Management, NEMA Research, Inc., Naples, USA; 2 Scientific Communications, NEMA Research, Inc., Naples, USA; 3 Pain Medicine, National Spine and Pain Centers, Rockville, USA

**Keywords:** a3 receptor, adenosine, adenosine receptors, anticancer drugs, cancer cell biology, cytoprotection, mast cells proliferation, tumor microenvironment (tme)

## Abstract

Found in all human cells, the purine nucleoside adenosine plays various roles in different metabolic pathways. Adenosine is not stored in vesicles but is released continuously, based on metabolic demands. Essential in energy production, adenosine is a full agonist at four known receptors (A1, A2A, A2B, and A3), and is produced either intracellularly or in the extracellular space. Adenosine is the building block for adenosine triphosphate (ATP), a vasodilator that inhibits certain cerebral neurotransmissions. Adenosine diphosphate (ADP) releases energy via its phosphate bonds and can “recharge” by adding phosphate groups later on, unlike ATP. The A3 receptors are most densely expressed in humans in the liver, lungs, immune cells, heart, and brain, and A3 agonists confer cytoprotection, making A3 agonists an intriguing potential anticancer drug. A3 receptors are so highly expressed in cancer cells and tumors that they serve as cancer biomarkers. To date, A3 agonists and A2A antagonists have emerged as potential anticancer drugs. Paradoxically, A3 is upregulated in primary tumors and metastatic disease, and A3 activity correlates with invasive actions of tumor cells. This contradictory effect, paralleled by the pro- and anti-inflammatory effects of A3, may be explicable because the downregulation of A3 receptors may produce different effects than A3 agonism. Adenosine is abundant in the tumor microenvironment (TME), and cancer cells seem adept at adjusting their metabolic processes to attune themselves to their specific TME. The derangement of energy metabolism is characteristic of cancer, and cancer spreads as normal cells in proximity to a tumor become increasingly neoplastic and tumorigenic. This expands our old notion of tumors as discrete, separate bodies and views them now as complex layers of cancer cells of different functions; these cells, which can include recruited normal cells, interact with each other. The role of mast cells is emerging as an important, albeit enigmatic, part of the TME. Mast cells are abundant in tumors and appear to have a pro-inflammatory effect, but it is not entirely clear if they serve to promote or oppose the growth of tumors. Crosstalk between mast cells and some types of cancer cells, involving adenosine, has been observed. The translational impact and clinical implications of these findings remain speculative.

## Introduction and background

Adenosine, a purine nucleoside, is found in every human cell and is a ubiquitous endogenous neuromodulator in other vertebrates. It plays a role in multiple different metabolic pathways [1]. However, adenosine is not stored in vesicles, but is released on a continuous basis in response to metabolic demands [2]. Adenosine is a full agonist at its four known receptors: A1, A2A, A2B, and A3; functional assays show that inosine may also be a partial agonist at A1 and A3 [3,4]. A1 and A3 receptors are coupled to G1 members of the G-protein family, while A2A and A2B are coupled to G2 members [4].

Although adenosine (chemical formula C_10_H_13_N_5_O_4_) has many functions, it plays an essential role in energy production [1,5]. Adenosine is also involved in the inflammatory process, hypoxia, and tissue injury [6]. Less clearly elucidated is emerging evidence that adenosine may have specific effects on cancer cells. Paradoxically, the activity at the A3 receptor can both promote and inhibit tumors. This duality of function may help elucidate the multiple mechanisms of cancer and could be a boon to more personalized strategies in medical oncology. The A3 receptor represents a potential new drug target.

The purpose of this narrative review is to summarize our limited, but growing, understanding of the protumorigenic and antitumorogenic properties of activity at the A3 receptor.

## Review

Methods

On June 18, 2025, the PubMed database was searched for (adenosine) AND (cancer) AND (A3), with results delimited to articles published in the past 10 years. This yielded 230 results. Google Scholar was searched the same day for “adenosine, cancer, A3,” and the first 20 results were taken. The Cochrane database was searched for “adenosine receptors, cancer,” and found no reviews, although there were a number of ongoing trials. Since these trials had not yet published any findings, there were no studies added from this search.

We included articles that were written in English, that dealt specifically with A3 receptors and cancer. Articles could be systematic reviews; systematic reviews and meta-analyses; narrative reviews; original studies (preclinical studies, clinical trials, randomized clinical trials, observational studies, and retrospective studies); case series; case reports; and editorials or correspondence. We excluded grey literature, and articles that were not available in English.

The bibliographies of these articles were searched as well. Articles were included if they offered insight into the A3 receptor and cancer care. This is a narrative and not a systematic review of a relatively new area of research.

Mechanistic biology of A3 receptors

Adenosine is a nucleoside composed of adenine (a purine) and ribose (a five-carbon sugar). Adenosine is a building block for adenosine triphosphate (ATP), which is a nucleotide consisting of an adenosine molecule with three phosphate groups attached as a chain to the ribose sugar. ATP functions as a vasodilator and acts as an inhibitory neurotransmitter in the brain [1]. In its phosphate-ribose bonds, the ATP molecule stores energy, which can be released with hydrolysis [1].

Adenosine diphosphate (ADP) has two phosphate groups and likewise releases energy via its phosphate bonds, but unlike ATP, ADP can “recharge” itself by adding back a phosphate group later [1]. Adenosine, ADP, and ATP are all composed of an adenine base and a ribose sugar, with the key differences among them being the number of attached phosphate groups. ATP may be considered the main - but not exclusive - source of energy within the cells (Table 1).

**Table 1:** Key distinctions among adenosine, adenosine diphosphate (ADP), and adenosine triphosphate (ATP) Adenosine, adenosine diphosphate (ADP), and adenosine triphosphate (ATP) are crucial energy sources to the cells and convert readily back and forth [1,7,8].

	Adenosine	ADP	ATP
Molecular structure	Adenine and ribose sugar	Adenine, ribose sugar, and 2 phosphate groups attached at the 5’ carbon of the ribose sugar	Adenine, ribose sugar, and 3 phosphate groups attached to the 5’ carbon of the ribose sugar
Phosphate groups	Single phosphate group attached to the ribose sugar (adenosine monophosphate (AMP))	Phosphate groups linked together	Phosphate groups in a chain, sometimes described as α, ϐ, and ϒ for the terminal phosphate
Energy	Low	Intermediate: When ADP is phosphorylated, it produces ATP	Acts as the cell’s “energy currency” with high-energy bonds that store significant amounts of potential energy
Functions	Found in DNA and RNA and can act as a signaling molecule	Plays a role in energy metabolism, blood clotting, and platelet aggregation. It is an intermediate to ATP	Main energy source for cellular processes, crucial in signal transduction
Conversion	Can be created from ADP via hydrolysis	Converts to ATP via phosphorylation (typically during cellular respiration). Can be hydrolyzed to AMP	Removal of 1 phosphate group releases energy and converts molecules to ADP

Adenosine is produced within the cell or in the extracellular space. While there is a much larger amount of intracellular versus extracellular adenosine, extracellular adenosine has particular clinical relevance [1,4]. Outside the cell, ATP can be broken down by ectonucleotidases (such as cluster differentiation (CD)39, which converts ATP to ADP/adenosine monophosphate (AMP), and CD37, which converts AMP back to adenosine). Adenosine is rapidly metabolized. Adenosine kinase can phosphorylate adenosine to AMP, which, upon further phosphorylation, produces ADP and ATP. This so-called “salvage pathway” allows cellular energy to be recycled. On the other hand, adenosine deaminase (ADA) converts adenosine to inosine, which can be metabolized to hypoxanthine, xanthine, and uric acid, whereupon it can be excreted from the body in urine [5,9].

Signal pathways and physiological implications

The therapeutic implications of adenosine, ATP, and ADP are vast, since their endogenous signaling pathways are numerous and varied [10]. To date, adenosine and its derivatives have been studied with respect to potential treatments for cardiovascular disease (in particular, arrhythmias and vascular disorders), seizures, central nervous system activities (including analgesia), muscle contractions, bone metabolism, inflammation, liver glucose metabolism, autism, and cancer [1,10]. Adenosine appears to have the potential to both promote and inhibit tumor growth and cancer cell proliferation, depending on context [11].

Adenosine receptors

All four known adenosine receptors have cytoprotective functions, but each of these receptors (designated as A1, A2A, A2B, and A3) has specific and distinct localizations and unique biochemical pathways [4,12]. In humans, the highest expressions of A3 receptors are localized in the liver, lungs, and immune cells, but there are substantial expressions found in the heart and brain as well [13,14]. The cytoprotective properties of A3 agonism have made this particular receptor a subject of interest as a potential anticancer drug target [12]. The A3 receptor binds to a biological target (ligand), yet it can be conjugated to a new, specific moiety [15]. Research suggests that conjugation decreases A3 selectivity, even creating dual ligands at A2A and A3 [15].

Two adenosine receptors, A2A and A2B, activate adenylate cyclase; conversely, A1 and A3 inhibit adenylate cyclase. The A2A receptor can inhibit the cytotoxicity of natural killer (NK) cells and T cells, and it promotes the production of other immune-suppressing cells, such as myeloid-derived suppressor cells (MDSCs) and regulatory T cells (Tregs). This suggests that A2A antagonism may be beneficial in cancer treatment [2].

The inhibition of adenylate cyclase reduces the amount of cyclic adenosine monophosphate (cAMP) in the cell, which in turn affects the phosphorylation of proteins involved in the mitogen-activated protein kinase (MAPK) and Akt signaling pathways [2].

Adenosine receptors are encoded by different genes, all of which belong to the G-protein-coupled receptor (GPCR) family [2]. The A1 and A3 receptors couple to the G-proteins of Gi, Gq, and Go; in contrast, the A2A and A2B receptors couple to Gs or Gq. Thus, adenosine may cause different functional outcomes depending on the receptor expression and the concentration levels of adenosine [16]. The levels of adenosine, along with the expression of the various adenosine receptors, can increase during times of stress [16].

All four adenosine receptors are expressed in cancer cells, but A3 receptors are particularly highly expressed in tumors and cancer cells, even in leukemia, lymphoma, melanoma, glioblastoma, and others [17]. A3 ligands reduce cancer cell proliferation and recruit other cells, notably NK cells, to help fight cancer [17].

On the other hand, the activation of A1, A2A, and A2B receptors is generally associated with pro-tumorigenic effects. While A2A, A2B, and A3 receptors can all be overexpressed in cancer cells, only the activation of A3 has been shown to protect against tumors, and only in certain cases [18]. For those reasons, both A3 receptor agonists and A2A receptor antagonists have emerged as promising targets for cancer treatment, in recognition of the interplay among tumor hypoxia, high levels of extracellular adenosine, and immune system suppression, all of which promote tumor growth [17]. Since these two receptors, A2A and A3, appear to play prominent roles in both the immune system and cancer progression, combination pharmacologic approaches may be considered for further investigation. Moreover, the A3 receptor has been recognized as a tumor marker, suggesting that adenosine receptor ligands may be present within tumors [17].

Selective agonists for all four adenosine receptors have been developed, but none of these agents has, at this time, received regulatory clearance to market [19]. The exception is adenosine, which is approved and indicated as an adjunctive agent to thallium-201 in myocardial perfusion scintigraphy for patients with exercise intolerance; it is not approved for use in cancer treatment [19].

It is important to note that the selectivity of the four adenosine receptors causes a concentration-dependent response to adenosine. When there is a high concentration of adenosine, all receptors are likely to respond, while low concentrations of adenosine may stimulate only the high-affinity receptors [6,20]. Adenosine concentration can also affect A3 expression in tissue [21]. This suggests that even subtle shifts in adenosine concentrations can have a marked impact on adenosine signaling, tumor progression, and staging [20].

The A2A and A2B adenosine receptors stimulate adenylyl cyclase (AC) and increase cAMP, while the A1 and A3 receptors do the opposite by inhibiting AC and decreasing cAMP levels [22]. A3 agonists are being considered in research as potential targets for anticancer drugs, but also for pharmacological treatments of rheumatoid arthritis, psoriasis, dry eye, glaucoma, and chronic hepatitis C [1]. Stimulation of the A3 receptors promotes neurite length by way of the PI3K/Akt pathway. This too holds therapeutic promise, in light of potential neuroprotection [22]. A3 receptor stimulation can trigger cytostatic activity and apoptosis [23].

Intracellular versus extracellular adenosine

Adenosine in the extracellular environment is derived from ATP, ADP, or hydrolysis of AMP by specific ectonucleotidases (ecto-nucleoside triphosphate diphosphohydrolase or CD39). By contrast, intracellular adenosine comes from AMP and S-adenosylhomocysteine hydrolysis by endo-5’-nucleotidase and SAH hydrolase, which can be converted to AMP by adenosine kinase or deaminated into inosine by ADA enzymes 1 or 2 [24]. It is also possible for the body to produce endogenous adenosine de novo [25].

The concentrations of adenosine in the extracellular versus intracellular environments are regulated by equilibrative nucleoside transporters (ENTs) and concentrative nucleoside transporters (CNTs) [26]. ENTs regulate adenosine concentrations in these two environments by passively allowing the movement of adenosine based on concentration differences, using the gradient created by sodium ions as the energy source. In a physiologic environment, adenosine would naturally shift from the extracellular to the intracellular environment, but under hypoxic conditions, ENT1 blocks this action and allows for the accumulation of higher concentrations of extracellular adenosine [26,27].

A3 enigma

In primary tumors and metastatic disease, A3 tends to be upregulated; the activation of A3 receptors has been linked to the migration of tumor cells and invasion. It has been shown that A3 agonism can stimulate melanoma and encourage the proliferation of colorectal cancer cells [28,29]. Conversely, inducing A3 receptors was able to inhibit colorectal and melanoma cell proliferation and was associated with apoptosis of lung cancer cells and hepatocellular carcinoma [30]. These contradictory findings have been explained by separating the function of downregulation of A3 receptors versus stimulation of the A3 receptors [31].

Likewise, A3 appears to exhibit both pro- and anti-inflammatory effects [32]. When adenosine binds to a receptor, it triggers a variety of actions: it activates the MAPK pathway, which plays a role in cell proliferation and differentiation [2]. A3 receptor activation is known to trigger pro-tumor activity in melanoma, glioblastoma, and colon cancer, but it exhibits antitumor activity in leukemia, lymphoma, sarcoma, prostate cancer, pancreatic cancer, breast cancer, and lung cancer [23]. Paradoxically, using in vitro models for melanoma and leukemia, a pro-apoptotic effect has been observed with adenosine [33]. It is speculated that this duality may be related to adenosine concentration, with low concentrations inhibiting tumor growth and higher concentrations conferring a pro-angiogenic effect [33].

Adenosine in the tumor microenvironment (TME)

Adenosine is particularly abundant in the TME, including even the surrounding vasculature, immune cells, fibroblasts, various signaling molecules, and the extracellular matrix itself [33-35]. Since most solid tumors create a hypoxic TME, adenosine levels tend to be high, and this, in turn, promotes angiogenesis in the tumor [33].

Tumor cells tend to metabolize glucose, lactate, pyruvate, hydroxybutyrate, acetate, glutamine, and fatty acids more rapidly than non-tumor cells [36]. Cancer biology is complicated by the fact that metabolism is so compartmentalized that variation and flexibility among tumor cells allow the production of ATP as an energy source while, at the same time, preserving reduction-oxidation (redox) balance. This diverts energy resources toward biosynthesis, which may advance tumor cell proliferation. In fact, it appears that cancer cells may adjust their metabolic processes in order to survive and proliferate in whatever tumor ecosystem they find themselves in [36]. The hypoxia typically observed in tumor cells leads to the production of high levels of adenosine, which then acts as an immunosuppressant [36].

Inhibition of adenosine pathways is an important target for anticancer treatments, and research has generally been directed toward reducing adenosine production in the tumor by targeting CD73 and CD39 and/or inhibiting adenosine signaling pathways by targeting the A2A and A2B receptors [37]. The cellular response to hypoxia involves the release of hypoxia-inducible factor 1 (HIF-1), which itself plays a role in angiogenesis and tumor cell metabolism [38]. Since both the expression of A3 receptors and the production of HIF-1 have clear associations, it is speculated that there are links between adenosine, hypoxia, and cancer that remain to be elucidated [39]. 

TMEs are a focal point for research into immunotherapeutic approaches for cancer. Cancer cells suppress the body’s natural immune responses; blunting the effect of natural immune cells permits the tumor cells to proliferate. Immunotherapies attempt to counteract these processes but have met with only limited clinical success, because natural resistance to these treatments is strong [37]. Further research is needed to develop more broadly effective immunotherapeutic approaches [18]. Adenosine can act as an immunosuppressant, particularly the extracellular stores of adenosine within the TME [18].

The derangement of energy metabolism is a hallmark of cancer. It develops when proliferative signaling can be maintained while, at the same time, cells can effectively evade or avoid growth suppressors, autophagy, and apoptosis enough to sustain angiogenesis [40]. Normal cells can progressively become increasingly neoplastic and take on these cancer characteristics, allowing them to develop into tumorigenic and even malignant cells. This expands our older notion of tumors as being separate, insular collections of cancer cells. Instead, it is now understood that tumors contain multiple types of cells, including recruited normal cells, and that these cells have potential interactions with each other. Thus, the term TME, recognized as a conglomeration of different types of cells, offers a more serviceable description of a tumor than the old concept [40].

Immunotherapy is changing cancer care, but immunotherapeutic interventions have limitations and are not particularly effective against solid tumors [41]. Combination therapy, in which conventional anticancer treatments such as radiation are used together with immunotherapy, has improved immunotherapeutic response rates [18,41]. Since extracellular adenosine is particularly abundant in the TME, it must be considered that adenosine may regulate certain cancer cell processes [6]. For that reason, adenosine-related therapeutic interventions have emerged as potential adjuncts to combination immunotherapeutic treatments [42,43].

Because they are overexpressed in cancer, A3 receptors are considered a biomarker for many cancer diagnoses, including melanoma, breast cancer, prostate cancer, liver cancer, pancreatic cancer, esophageal cancer, and lung cancer [29,44]. Peripheral blood cells taken from human colorectal cancer patients demonstrate the overexpression of A3 [29].

Extracellular environment

ATP is ubiquitous in the extracellular environment, although a healthy metabolic system strictly regulates the quantity of extracellular ATP to low levels in the nanomolar range. Extracellular ATP can increase to micromolar levels in the presence of various factors, including tumorigenesis and inflammation [18]. Extracellular ATP can be a red flag, while extracellular adenosine generally confers anti-inflammatory, cytoprotective, and immunosuppressive effects [30]. Extracellular adenosine production is mediated by the cell surface ectoenzymes CD73, CD39, and CD38 [30]. The process can involve extracellular ATP, which is hydrolyzed to ADP and AMP; AMP is then converted to extracellular adenosine by CD73 and CD39. When high levels of ATP are present, and CD39 and CD73 are overexpressed, extracellular adenosine can accumulate.

Cancer cells may have certain genetic mutations that facilitate altered purine metabolism, allowing increased production of extracellular adenosine or reduced degradation of extracellular adenosine [18]. Extracellular adenosine can be eliminated by ADA, converting it to inosine or by nucleoside transporters that carry extracellular adenosine into the cell for conversion into AMP by adenosine kinases [5].

Inflammasomes are large protein complexes capable of launching an inflammatory response when they detect pathogenic or even exogenous danger. This triggers a downstream inflammatory response via caspase signaling cascades. The NLRP3 inflammasome plays a crucial part in the TME because it mediates inflammation, immune evasion, angiogenesis, and proliferative signaling pathways (which can facilitate tumor growth), while, at the same time, inhibiting apoptosis [45,46]. ATP has been considered a damage-associated molecular pattern (DAMP) and is capable of activating the NLRP3 inflammasome by way of the purinergic receptors associated with adenosine [47].

The conversion of extracellular ATP into extracellular adenosine encourages the progression of tumors and facilitates tumor escape from antitumor immunity [11]. Extracellular adenosine is produced mainly by ectonucleotidases CD39 and CD73; other pathways likely exist but are not well understood. Activation of A2A and A2B receptors suppresses the antitumor activity of immune cells [11,18].

Role of mast cells

Mast cells originate from committed progenitors in the bone marrow, passing from the marrow into the vascular space and, from there, entering tissue, where they mature and differentiate [21]. The TME is inhabited to a large extent by mast cells, which contribute to cancer progression [16]. Tumors may be considered multicellular "organs" that infiltrate non-tumor cells and incorporate them for their own survival, growth, and metastases. These infiltrators are mainly immune system cells, possessing both pro-tumor and anti-tumor effects [31].

The role of mast cells in cancer is less clear. When stimulated, mast cells release pro-inflammatory mediators. The abundance of mast cells in tumors has emerged as a controversy, because it is disputed whether mast cells promote or restrict tumor growth [16]. The tumor response of mast cells has even been described as “plastic” - that is, variable [16].

The A3 receptor appears to play a role in mast cell activation [16]. Mast cell activation by pancreatic-cancer-cell-derived membranes has been shown to be mediated in part by the A3 receptor [31]. When these mast cells are activated by pancreatic-cancer-cell-derived membranes, interleukin (IL)-8 is released [31]. Thus, the role of mast cells in cancer is perplexing, as evidence shows they may have simultaneous pro- and anti-growth effects on tumors, likely related to the TME [16].

Mast cells have been studied in a variety of inflammatory disorders, including cancer, and play a role in both acquired and natural immunity [48]. Mast cells possess the ability to selectively release pro-inflammatory mediators without degranulation [48]. Crosstalk between mast cells and certain cancer cells has been established, and this communication involves adenosine [20]. Mast cells migrate to the TME, releasing certain factors while, at the same time, being activated by cancer cells to produce adenosine [16]. This adenosine signaling process is mediated by A3, releasing angiogenic factors and cytokines such as IL-8 that may contribute to tissue remodeling, as well as vascular endothelial growth factor, amphiregulin, and secreted phosphoprotein - all of which facilitate tumor growth and progression [16].

Mast cells stimulate the migration of MDSCs toward the tumor, causing these cells to produce IL-17, which, in turn, attracts Tregs and induces them to produce the enigmatic cytokine IL-9 [20,49]. This cascade, culminating in IL-9 production, appears to promote the survival of pro-tumor mast cells in the TME. There is a closed-loop communication within the TME among mast cells, MDSCs, and Tregs [20].

Clinical implications and discussion

As our understanding of the molecular mechanisms of A3 receptors expands, clinical and translational implications for oncology emerge. Based on our current understanding, it appears that adenosine and the A3 receptors play an important role in certain - possibly all - cancers. The ability to modulate adenosine levels via the A3 receptors is currently an important topic for investigation [23]. A number of drugs are in development, and some have shown promise in preclinical studies [23], but none have yet been approved for market, and important differences between cancer in humans and rodents must be acknowledged [21].

Adenosine can be very challenging to study because the half-life of adenosine in humans is less than 10 seconds. Delivery systems for any adenosine drug must be able to facilitate more sustained action [1]. Another challenge is that measuring central nervous system adenosine levels in humans is not always practical, and peripheral levels of adenosine do not always provide clinically relevant information [50].

Perhaps the most formidable challenge to the study of A3 as a therapeutic target in oncology is the fact that it plays seemingly “contradictory” roles in tumorigenesis and cancer cell proliferation. Further research is urgently needed, particularly with regard to how A3, immune cells, stromal cells, mast cells, and endothelial cells interact. For instance, an A3 antagonist has been evaluated in an in vitro study against human prostate cancer [51]. Yet, other research toward the same objective of treating cancer is working on A3 agonists [2].

The ability to selectively target A3 receptors represents a significant advance in oncology and personalized medicine, because this is the first target that offers dual benefits: anticancer benefits, plus support of a healthy immune system [17]. Namodenoson is perhaps the leading A3 agonist in clinical development [17]. Other A3 agonists include preladenant (MK-3814 or PBF-509), CP1-444, and AZD4635 [17]. A phase I study in healthy volunteers found Namodenoson was safe and well-tolerated. A subset of patients in a phase I/II trial demonstrated preliminary proof that Namodenoson offered anticancer action in patients with advanced Child-Pugh B (CPB) hepatic dysfunction. Median survival in this study was 8.1 months [19,52]. A randomized, blinded, placebo-controlled study with advanced hepatocellular carcinoma or CPB patients found Namodenoson was not superior to placebo in overall survival; however, a subgroup of patients evidenced a significant increase in progression-free survival at 12 months (44% vs. 18%, p = 0.028) [19,53].

This article has several limitations. First, it is a narrative, rather than a systematic, review and attempts to provide a broad narration of the topic rather than a deep dive into specific areas. An inherent risk in all narrative reviews is that there may be bias or omissions. This is a new field of active research, meaning that information may change rapidly.

## Conclusions

Adenosine receptors (A1, A2A, A2B, and A3) are being elucidated for their myriad roles in energy production and metabolic processes. In particular, the relationship between A3 receptors and cancer cells has proven both enigmatic and important. A3 receptors are abundant in cancer cells, tumors, and the TME, to the extent that they are useful as cancer biomarkers. A3 receptors may play a role in the activation of mast cells, and the inhibition of adenosine pathways may be a target for anticancer treatments. However, cancer biology is complicated by the fact that metabolism in tumor cells is compartmentalized and dynamic, with cancer cells adjusting their metabolic processes to the specific TME. The fact that activity at the A3 receptor can both promote and inhibit tumorigenesis offers an intriguing duality of function, parallel with the fact that A3 activity can be both pro- and anti-inflammatory. Further research may help elucidate the mechanisms of A3 and other adenosine receptors and open avenues to more personalized oncologic strategies.
